# Systems biology: An emerging strategy for discovering novel pathogenetic mechanisms that promote cardiovascular disease

**DOI:** 10.21542/gcsp.2016.27

**Published:** 2016-09-30

**Authors:** Bradley A. Maron, Jane A. Leopold

**Affiliations:** 1 Division of Cardiovascular Medicine, Department of Medicine, Brigham and Women’s Hospital and Harvard Medical School, Boston, MA, USA; 2 Department of Cardiology, Boston VA Healthcare System, Boston, MA, USA

## Abstract

Reductionist theory proposes that analyzing complex systems according to their most fundamental components is required for problem resolution, and has served as the cornerstone of scientific methodology for more than four centuries. However, technological gains in the current scientific era now allow for the generation of large datasets that profile the proteomic, genomic, and metabolomic signatures of biological systems across a range of conditions. The accessibility of data on such a vast scale has, in turn, highlighted the limitations of reductionism, which is not conducive to analyses that consider multiple and contemporaneous interactions between intermediates within a pathway or across constructs. Systems biology has emerged as an alternative approach to analyze complex biological systems. This methodology is based on the generation of scale-free networks and, thus, provides a quantitative assessment of relationships between multiple intermediates, such as protein-protein interactions, within and between pathways of interest. In this way, systems biology is well positioned to identify novel targets implicated in the pathogenesis or treatment of diseases. In this review, the historical root and fundamental basis of systems biology, as well as the potential applications of this methodology are discussed with particular emphasis on integration of these concepts to further understanding of cardiovascular disorders such as coronary artery disease and pulmonary hypertension.

## Introduction

The current era of human health and disease research is defined, in part, by an explosion of technology that permits the measurement of biological data on a grand scale^1^. Increased availability, cost-effectiveness, and access to gene chip arrays, whole genome sequencing, metabolomic platforms, and next generation RNAseq among other approaches has resulted in the application of these methodologies to profile the genetic and molecular signature of virtually all biological mediums^2,3^ across normal and disease states alike^4^. The magnitude of data derived from these methods, which in one recent epigenetic study of myocardial infarction (MI) included an analysis of the protein-coding regions for 9,793 patient genomes and single nucleotide variants in 64,132 samples^5^, and the accelerating rate by which these methods are utilized in research have outpaced the evolution of analytical strategies needed for their optimal interpretation. Specifically, approaches that rely heavily on reductionism, such as association studies, cannot account adequately for the complexity inherent to datasets of this scale^1^.

**Figure 1.**
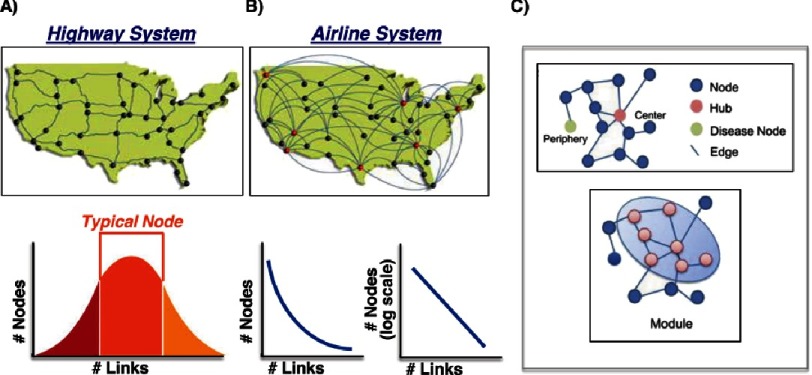
Network topography. (A) In a random network, the majority of nodes shares a similar number of links, and is analogous to the United States highway system in which cities are represented as nodes and interconnecting roads are represented as links. (B) In non-random networks, such as in biological systems, and, as is represented in this illustration the hub-spoke system of air travel patterns, a minority of nodes are highly connected. (C) This characteristic establishes a hierarchy based on node connectivity, which is grouped by convention in ascending order of complexity as: node, hub, module and neighborhood. These levels of connectivity, in turn, may be topographical within a network, indicate a common biological function, or grouped according to association with diseases. Figure adapted from^6,37^ with permission pending.

Network theory, in turn, leverages mathematical modeling to quantify multiple, simultaneous interactions between variables (e.g., interactome). This method allows for the graphical depiction of relationships across a range of subcellular components (e.g., protein-protein, mRNA-protein, miRNA, others), and, therefore, is not limited by a particular cell type, species, or pathway ^6^. Organizing networks further according to connectivity patterns provides a hierarchical basis of the associations within parts of a pathway or between pathways (Figure 1). Enriching networks by integrating gene sets linked to a specific pathophenotype may be used to narrow assessments of interaction clusters^7^. Overall, these strategies allow for a robust, but structured approach to integrating, depicting, and analyzing big data for the purposes of understanding better the interactions that underlie complex pathophenotypes.

## Historical perspective: Reductionism in science

The French philosopher, mathematician, and physicist René Descartes is often referred to as the father of reductionism, based largely on his “Rules for the direction of the mind” written originally in 1628^8^, which posited 12 rules of scientific methodology. Among them, rules V and VI stipulated problem resolution is best achieved through ‘intuitive apprehension of all those that are absolutely simple’ and if ‘obscure propositions are reduced step by step to those that are simpler.’ Descartes applied this rationale to his workings that would ultimately serve as the basis for contemporary geometry, algebra, and physics. In fact, this form of deductive, rule-based reasoning was utilized by many of the great scientists and meta-scientists from the classical and contemporary eras, including Galileo Galilei (astronomy; 1564–1642), Sir Isaac Newton (physics; 1643–1727), Immanuel Kant (philosophy, 1724–1804), and, perhaps most relevant to biology, Sir Charles Darwin in his theory of natural selection (1809–1882).

**Figure 2.**
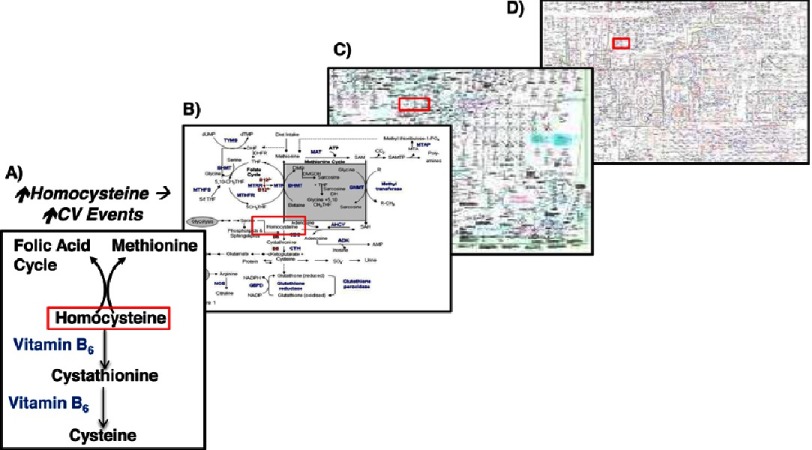
The biochemical pathway regulating homocysteine metabolism presented to scale within global mammalian metabolism. Despite numerous basic, translational, and epidemiological scientific reports establishing a link between hyperhomocysteinemia and cardiovascular disease risk, folic acid/B-vitamin supplementation to reduce plasma homocysteine levels is not associated with improved outcome in clinical trials. (A) Mechanistic data demonstrating the beneficial effects of folic acid/B-vitamins on homocysteine-induced vascular dysfunction are derived largely from studies focusing on factors directly relevant to homocysteine biochemistry. (B–D) However, this reductionist approach does not account for the consequences of folic acid or B-vitamins on off-target pathways that are connected to homocysteine and include intermediaries that regulate cell proliferation and survival. To consider the effects of folic acid/B-vitamins on homocysteine metabolism and other pathways, a systems level approach is required.

In the latter part of the 20th century, however, some challenged the appropriateness of law-based theory to biological sciences^9^. The reasoning for this skepticism was based, in part, on the identification of the following dilemmas: (i) reductionism implies that rules affect all components of a system similarly, which is unlikely to be the case in living organisms *in vivo*, and (ii) the sole focus of an analysis on fundamental components of a biological system may not be possible or warranted. For example, assessing phenotype expression according to genetic variability alone does not account for post-transcriptional regulation of RNA or post-translational modification of proteins, despite the staggering frequency of these events reported in proteome studies^10^ and the importance of these regulatory mechanisms in determining physiologic and disease states^11–13^. Furthermore, reductionist methodologies may have a number of important unintended consequences in applied experimental biology. By narrowing the scope of study solely to a single pathway or specific interaction between two intermediaries, the effects of perturbations to the larger system are often unaccounted for (or overlooked). This, in turn, has important ramifications when considering the translational relevance of a drug or effector from studies *in vitro* to investigations *in vivo* or clinical trials. It has been proposed, for example, that this principal may underlie the failure of folic acid and B-vitamin supplementation to abrogate the cardiovascular risk associated with hyperhomocysteinemia^14^ despite numerous epidemiologic reports in patients supporting mechanistic data demonstrating that folic acid treatment limits the adverse effect of homocysteine on endothelial dysfunction, atherogenesis, and thrombosis^15^. In these studies, however, the pro-proliferative, tumorigenic, and diverse metabolic effects of folic acid/B-vitamins were not generally considered^16^ (Figure 2). Thus, the possibility remains that those or other untested (and potentially detrimental) consequences of these therapies may account for the failure of folic acid/B-vitamin therapy to improve outcome in randomized clinical trials^17^.

## Network theory in applied biological sciences

In random networks, the density of links (e.g., connections) between nodes (e.g., entities) follows a Poisson (normal) distribution (Figure 1A). In this configuration, the majority of nodes share a similar degree of connectivity, and highly connected nodes are not common^6,18^. However, in biological networks (among other naturally occurring network types), the relationship between nodes and links appears to be quite different. Jeong and colleagues^19^ constructed a protein-protein interactome based on *S. cerevisiae* proteome data and demonstrated the probability that a given protein interacts with other proteins follows a power law, which was a finding that was confirmed in a bacterium protein interactome, among other biological interactomes^20^. That is, a few nodes share the property of high connectivity, while a greater number of nodes share the property of low connectivity. These observations suggested that the relationship between nodes and link density in biological networks is not stochastic, but is based on non-random order. Indeed, subsequent work has demonstrated an inverse relationship between the number of nodes and the number of links associated with each node indicating that there is hierarchical order of connectivity with respect to function for nodes within a biological network^19,21^. Furthermore, the number of links correlates positively with the biological importance of a node, as removal of highly connected nodes corresponds to an increase in organism lethality^19^. This concept can be expressed by *betweeness centrality*, which reflects the number of shortest paths through a given node and is used to identify important or essential nodes within a network^22,23^. While this may seem to suggest that gene mutation (i.e., node deletion) is the principal driver of phenotype expression, this is, in fact, an uncommon occurrence^24^. By contrast, network perturbation that occurs through disruption of connectivity pathways via transcriptional, post-translational, and environmental effects is more likely to account for expression of complex diseases^25–27^.

Peri and colleagues constructed the first human protein interactome by establishing 24,385 binary protein-protein interactions, which was based on manual curation of the literature describing physical interactions between proteins discovered primarily from *in vitro* and *in vivo* experiments^28^. The same group later supplemented this database with novel protein-protein interactions derived from several additional protein databases^29–31^. They next analyzed the relationship between protein interactions according to subcellular compartment and their relative proximity within the network. They observed differences in connectivity patterns according to organelle and within specific biochemical pathways^32^. These findings contributed to a larger set of observations indicating that levels of organization within networks exist based on common biochemical, cellular, functional, and phenotypic threads^33–36^.

Subsequently, nomenclature for grouping based on ascending order of clustering scale has been proposed: node<hub<module<neighborhood, in which hubs represent highly connected nodes, modules define groups of highly connected nodes in close proximity to one another, and neighborhoods represent locally densely connected modules relative to the larger interactome^37,38^. These associations are commonly topographical (within the network), functional (in association with a common biochemical pathway), or disease-associated (related to a particular pathophenotype)^6^.

**Figure 3.**
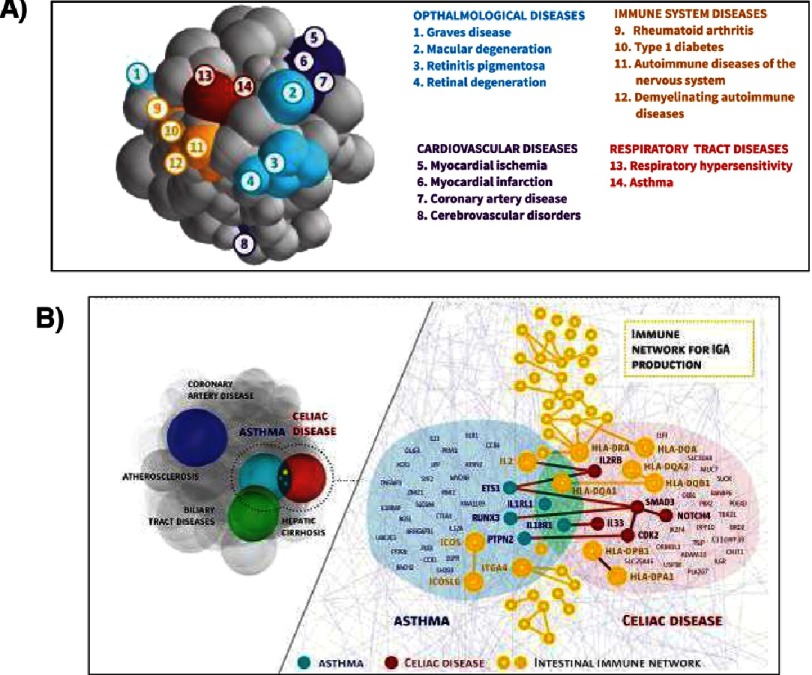
(A) A 3-dimensional representation of the relationship between diseases in the consolidated human interactome by Meche and colleagues^39^. In this analysis, the physical distance between diseases is proportional to the shortest distance between closely connected protein pairs across disease modules. Diseases whose modules (spheres) overlap are predicted to have a common molecular pathobiology. (B) This methodology identified unexpected overlap between certain diseases, such as hepatic cirrhosis, asthma, and celiac disease (*left*). A network-level map demonstrates the biological/immunological basis of overlap between in the asthma-celiac disease network-neighborhood. Figure and legend reproduced with some modifications from^39^ with permission pending.

Recently, Menche and colleagues^39^ focused attention on the validation of disease modules within the *consolidated human interactome*, which is a collection of all biologically relevant molecular interactions^35,39–41^. They compiled 141,296 physical interactions between 13,460 proteins derived from experimental data involving protein-protein interactions and interactions along metabolic and kinase-substrate pathways. They next analyzed these data according to the relationship between pathways and 299 human diseases. By developing a mathematical code to abridge deficiency in the pool of known disease-associated genes, the investigators were able to identify disease modules while acknowledging that the current version of the consolidated human interactome remains incomplete (Figure 3). They observed that the position of two disease modules in this analysis was not random and, in fact, predicted biological and clinical similarities based on overlap between modules for gene ontology, gene co-expression, clinical symptoms, and probability of disease co-morbidity occurrence^39^. Taken together, these findings validate the translational relevance of molecular interaction mapping, illustrate the biological continuum spanning related pathophenotypes, and place into close proximity diseases considered previously to be unrelated (e.g., glioma and myocardial infarction).

## Systems biology in cardiovascular medicine

### Coronary artery disease

Reports leveraging systems biology to study the basis of human disease are populating the medical literature at an accelerating rate^42^, and include reports on chronic obstructive pulmonary disease^43^, solid tumor cancer^44^, gastrointestinal disorders^45^, and pharmacotherapeutics^46^ among other areas^47^. Most relevant to cardiovascular medicine, systems biology has been used to decipher the interactions between pathways that regulate inflammation, cell proliferation, apoptosis, thrombosis, and fibrosis in relation to diseases of the myocardium, pulmonary circulation, and systemic vasculature.

For example, Liu and colleagues performed a gene co-expression network analysis recently using publically available microarray data acquired from peripheral blood samples in patients with coronary artery disease (CAD)^48^. In this analysis, 3711 differentially expressed genes were segregated into 21 modules, and glucose-6-phosphate dehydrogenase (G6PD) emerged as a highly connected node within the principal module. These data, which depict G6PD as an important hub in the pathogenesis of CAD, are in support of prior experimental data implicating deficient levels of this antioxidant enzyme as a cause of endothelial dysfunction, vascular stiffness, and systemic hypertension^49–51^. In related work from the Stockholm Atherosclerosis Gene Expression (STAGE) study, unbiased two-way clustering analytics performed on mRNA data from visceral fat and atherosclerotic lesions from patients was used to predict CAD severity^52^. The transendothelial migration of leukocytes (TEML) pathway as well as the LIM domain binding 2 protein emerged from this analysis as important targets linked to clinically expressed CAD, although confirmatory experimental data in support of these findings remain forthcoming.

Others have utilized networks for the purpose of determining prognosis following MI^53,54^. The ‘my-inflamome,’ for example, is an interactome that included 2,595 proteins and 6,181 interactions based on seed genes related to inflammatory biomarkers used in the clinical management of MI patients^55^. From this, 21 highly-interconnected modules were described according to their association with key biological processes (e.g., post-translational protein modification, apoptosis, etc.). Combinations of modules and highly connected nodes were entered into supervised computational and logistical regression analyses to identify Tissue Necrosis Factor-α associated factor (*TRAF2*), SHKBP binding protein-1 (*SHKBP1*), and Ubiquitin B (*UBC*) as genes that may prognosticate clinical outcome (defined as favorable [LVEF >40% post-MI] vs. unfavorable [LVEF <40% post-MI]).

Systems approaches are also being increasingly utilized to explore novel pathogenetic mechanisms underlying primary cardiomyopathies, including fibroblast-myocyte interactions^56^, the transcriptomic basis for cardiomyocyte remodeling^57^, biomarker identification in viral myocarditis^58^, and the proteomic basis of ischemic and reperfusion myocardial injury^54,59^. In some cases, systems biology has been leveraged to critically analyze data derived from genome-wide association studies (GWAS) reporting genetic variants in cardiomyopathy. For example, Backes and colleagues^60^ reviewed data on ∼280,000 gene variants from 909 dilated cardiomyopathy patients and 2,120 controls, and identified enrichment pathways (using the Kyoto Encyclopedia of Genes and Genomes [KEGG] database) containing variants that were highly statistically significant between patient groups. From this analysis, focus was directed to nucleotide excision repair-related pathways. In particular, RAD23B, which regulates DNA damage recognition and base excision repair, emerged as a candidate gene in this analysis. However, the relevance of RAD23B or other genes implicated in this study to cardiomyopathy requires further validation experimentally.

### Pulmonary vascular disease

Pulmonary hypertension is defined currently by a mean pulmonary artery pressure ≥ 25 mmHg assessed by cardiac catheterization performed supine at rest^61^, and often occurs in the setting of left heart disease, parenchymal lung disease, or thromboembolism. Rarely, patients with PH that occurs due to interplay between molecular and genetic factors in the absence of other comorbidities are reclassified as having pulmonary arterial hypertension (PAH)^62^. In clinical practice, however, overlap between pulmonary hypertension sub-groups is common, which may confound diagnosis and treatment decision-making^63,64^. Therefore, there is an increasing emphasis on studying the molecular basis of pulmonary hypertension in order to delineate pulmonary vascular from other forms of cardiopulmonary diseases^65,66^, as well as to predict treatment-responsiveness clinically^67^.

**Figure 4.**
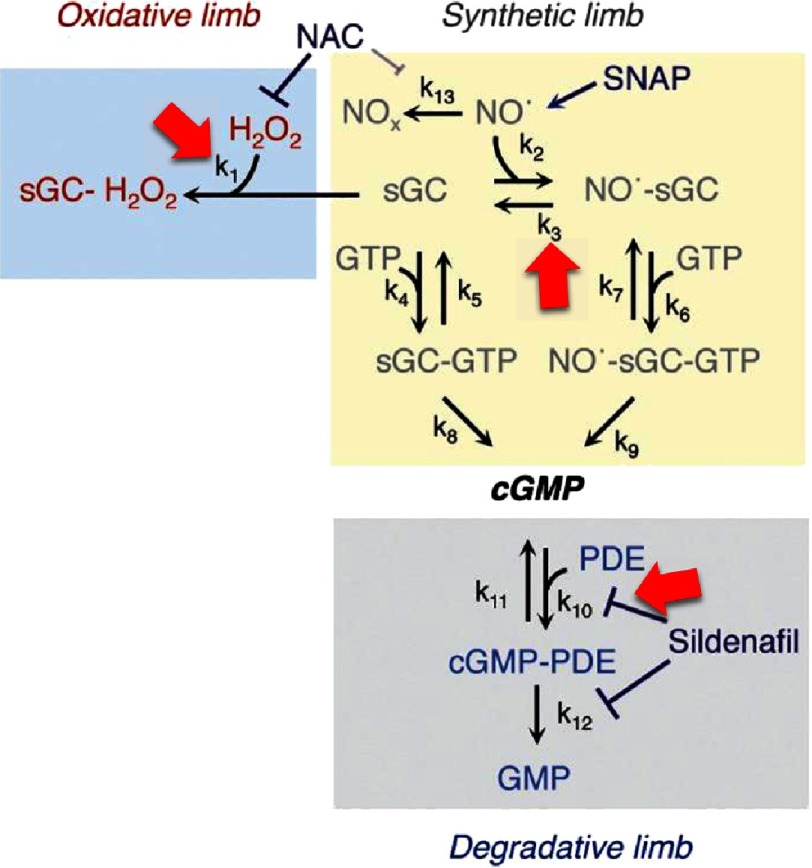
Nitric oxide-cGMP signaling. The vasodilator and anti-mitogenic molecule nitric oxide (NO) may be synthesized endogenously by upregulation of NO synthase enzymes or enzymatic conversion from nitrite, as well as exogenously by pharmacotherapeutic NO donors. The principal NO target in vascular cells is the heterodimeric enzyme soluble guanylyl cyclase (sGC), which converts GTP to the second messenger cGMP. Under conditions of increased oxidant stress, sGC may be oxidized to inhibit sGC sensing by NO that impairs normal NO-dependent vascular smooth muscle cell relaxation. Alternatively, cGMP may be hydrolyzed by phosphodiesterases to counter-regulate NO bioactivity. Predictions using a systems approach that considered reaction kinetic combinations for various steps in this biochemical pathway identified *k*_1_, *k*_3_, and *k*_10_ (red arrows) collectively as an optimal target to modulate NO-cGMP signaling. cGMP, cyclic guanosine 3′, 5′-monophosphate; GMP, guanosine-5′-monophosphate; GTP, guanosine-5′-triphosphate; H2O2, hydrogen peroxide; NAC, N-acetylcysteine; NO^.^, nitric oxide; NOx, oxidized (inactive) nitrogen oxides; PDE, phosphodiesterase; SNAP, S-Nitroso-n-nacetylpenicillamine; sGC, soluble guanylyl cyclase. Adapted with some modifications from^71^ with permission pending.

Systems biology has been utilized to characterize networks that regulate pulmonary vascular remodeling and the development of PAH. Activation of soluble guanylyl cyclase (sGC) by nitric oxide (NO) stimulates the conversion of GTP to cGMP, which, in turn, is a potent antimitogenic second messenger that promotes vascular smooth cell relaxation. Hydrolysis of cGMP to inactive 5’-GMP by phosphodiesterase type-V (PDE-V) is an endogenous counter-regulatory mechanism controlling NO bioactivity. However, diminished NO bioavailability and impaired NO-sGC signaling due, in part, to increased pulmonary vascular oxidant stress^68^ are associated with endothelial dysfunction, increased vascular smooth muscle cell proliferation, thrombosis, and vascular fibrosis that contribute to adverse vascular remodeling and pulmonary hypertension in PAH^63^. Although PDE-V inhibition and other novel direct NO delivery systems^69^ provide benefit to PAH patients^70^, the optimal strategy for inducing maximal NO bioactivity is not established. By modeling each step of the NO-sGC-cGMP reaction (12 molecular species and 12 rate constants), however, the effect of oxidation on single reactions, reaction pairs, or reaction trios was calculated recently to address this issue with respect to the NO-sGC-cGMP pathway in pulmonary artery smooth muscle cells (Figure 4). This approach identified sGC oxidation, NO dissociation from sGC, and PDE-mediated hydrolysis of cGMP as a collective is the principal enzymatic regulator of NO bioactivity *in vitro*, and sheds important new light on the potential value of a systems approach to pharmacology in cardiopulmonary disease^71^.

Systems biology has also been used to identify novel pathogenetic mechanisms controlling vascular remodeling in PAH. Parikh and colleagues generated a pulmonary hypertension network by mapping 131 published genes associated with the development of pulmonary hypertension to the consolidated human interactome^72^. An algorithm-based approach was then used to predict miRNA regulation of the PH-associated genes, which allowed for rank-ordering of 29 miRNA groups based on their anticipated regulation of pulmonary hypertension-relevant pathways, which in this analysis involved gene sets associated with hypoxia, inflammation, and bone morphogenetic protein-signaling. The investigators identified miR-21 targeting of Rho/Rho kinase from this approach as potentially relevant to pulmonary hypertension. This prediction was then validated by demonstrating miR-21-dependent upregulation of Rho/Rho kinase in cultured pulmonary endothelial cells, which corresponded to impaired endothelial nitric synthase expression *in vitro* and vascular remodeling and pulmonary hypertension *in vivo*. Similar approaches have been utilized to understand further mechanisms underpinning the fibrotic and the contractile vascular phenotype characteristics of PAH^73–75^.

## Conclusions

The current era of scientific investigation, in which large-scale datasets are used to identify novel genetic or molecular etiologies for disease expression, has brought to light some limitations in the application of association studies and other reductionist analytical methods. Network theory is an evolving strategy that utilizes mathematical modeling to illustrate simultaneously numerous interactions between subcellular components. Discovering and reporting relationships based on hierarchical levels of connectivity assists in stratifying network nodes according to importance, and provides a scale-free model by which to pursue unexpected targets involved in disease pathobiology or treatment. Currently, systems biology methods have been reported across various areas within cardiovascular disease research, including coronary artery disease and pulmonary hypertension. Coupling this and other similar approaches with experimental methods to validate network findings is likely to increase the translational potential of observations *in silico* to clinically relevant findings for patients affected by cardiovascular disease.

## Competing Interest

B.A.M.: Investigator initiated research supported by Gilead Sciences, Inc.

## Funding Sources

This work was supported by 1K08HL11207-01A1, American Heart Association (AHA 15GRNT25080016), Pulmonary Hypertension Association, Cardiovascular Medical Research and Education Fund (CMREF), and Klarman Foundation at Brigham and Women’s Hospital.

## Author Contributions

Bradley A. Maron and Jane A. Leopold contributed to the drafting of the manuscript and reviewed and revised it for critical content, and have given final approval to the manuscript version submitted for publication.

## ABBREVIATIONS

miRNA: microRNA
MI: myocardial infarction
G6PD: glucose-6-phosphate dehydrogenase
CAD: coronary artery disease
KEGG: Kyoto Encyclopedia of Genes and Genomes
GWAS: genome-wide association study
PAH: pulmonary arterial hypertension
NO: nitric oxide
PDE: phosphodiesterase
cGMP: Cyclic guanosine monophosphate
GMP: guanosine triphosphate
sGC: soluble guanylyl cyclase
